# Comparative transcriptome, physiological and biochemical analyses reveal response mechanism mediated by *CBF4* and *ICE2* in enhancing cold stress tolerance in *Gossypium thurberi*

**DOI:** 10.1093/aobpla/plz045

**Published:** 2019-08-09

**Authors:** Xiaoyan Cai, Richard Odongo Magwanga, Yanchao Xu, Zhongli Zhou, Xingxing Wang, Yuqing Hou, Yuhong Wang, Yuanming Zhang, Fang Liu, Kunbo Wang

**Affiliations:** 1 State Key Laboratory of Cotton Biology /Institute of Cotton Research, Chinese Academy of Agricultural Science, Anyang, Henan, China; 2 College of Plant Science and Technology, Huazhong Agricultural University, Wuhan, Hubei, China; 3 School of Biological and Physical Sciences (SBPS), Jaramogi Oginga Odinga University of Science and Technology (JOOUST), Bondo, Kenya

**Keywords:** Cold tolerance, gene expression, *G. thurberi*, signalling pathways, transcriptome

## Abstract

Low temperature is one of the key environmental stresses that impair plant growth and significantly restricts the productivity and spatial distribution of crop plants. *Gossypium thurberi*, a wild diploid cotton species, has adapted to a wide range of temperatures and exhibits a better tolerance to chilling stress. Here, we compared phenotypes and physiochemical changes in *G. thurberi* under cold stress and found this species indeed showed better cold tolerance. Therefore, to understand the molecular mechanisms of the cold tolerance in *G. thurberi*, we compared transcription changes in leaves of *G. thurberi* under cold stress by high-throughput transcriptome sequencing. In total, 35 617 unigenes were identified in the whole-genome transcription profile, and 4226 differentially expressed genes (DEGs) were discovered in the leaves upon cold treatment. Gene Ontology (GO) classification analyses showed that the majority of DEGs belonged to categories of signal transduction, transcription factors (TFs) and carbohydrate transport and metabolism. The expression of several cold-responsive genes such as *ICE1*, *CBF4*, *RAP2-7* and abscisic acid (ABA) biosynthesis genes involved in different signalling pathways were induced after *G. thurberi* seedlings were exposed to cold stress. Furthermore, cold sensitivity was increased in *CBF4* and *ICE2* virus-induced gene silencing (VIGS) plants, and high level of malondialdehyde (MDA) showed that the *CBF4* and *ICE2* silenced plants were under oxidative stress compared to their wild types, which relatively had higher levels of antioxidant enzyme activity, as evident by high levels of proline and superoxide dismutase (SOD) content. In conclusion, our findings reveal a new regulatory network of cold stress response in *G. thurberi* and broaden our understanding of the cold tolerance mechanism in cotton, which might accelerate functional genomics studies and genetic improvement for cold stress tolerance in cultivated cotton.

## Teaser

Cotton is an important crop and the primary source of natural fibre for the textile industries, and is thus often referred to as white gold. Its production has continued to decline due to the effects of various abiotic stresses, occasioned by narrow genetic diversity and intensive selection. Utilization of the wild cotton germplasm is poised to unravel the problem of abiotic stress and the increase in diversity of the cultivated cotton. Transcriptome analysis and RNAi technique revealed the integral role played by two novel genes, *CBF4* and *ICE2* in enhancing cold stress tolerance in cotton.

## Introduction

Low temperature is one of the key environmental stresses, which drastically impairs plant growth and significantly restricts the productivity and spatial distribution of crop plants (Zhou *et al.* 2011). There are two types of the low temperature stress which affect plant growth and development, namely chilling (0–20 °C) and freezing, which occurs when the temperature falls below zero (Song *et al.* 2015). With the effects of global warming and drastic weather changes, the low temperature has become one of the major forms of abiotic stress with enormous effects on plant growth and development (Hasanuzzaman *et al.* 2013). The low temperature of the field environment restricted the plant’s vegetative growth and delayed all the phenological stages in comparison to plants grown under controlled environment (Barrero-Gil *et al.* 2016). Apart from this, it led to some vegetative aberrations like chlorosis, necrosis of leaf tips and curling of whole leaves. The damage to the reproductive stage involved the abscission of juvenile buds, flowers, abortion of bolls and in extreme conditions that cause plant death (Bolek 2010). Plants have developed several response mechanisms to various forms of abiotic stressors, including chilling and freezing stresses. The most common phenomenon is acclimation, which mainly occurs through enhanced tolerance to environmental stress after priming treatment (Shi *et al.* 2018). The plant stress response is regulated at the molecular level (Iba 2002) and induces several changes in biochemical pathways and physiological processes of stressed plants (Bartwal *et al.* 2013). Generally, plants from temperate regions, including *Arabidopsis*, winter wheat, barley and oilseed rape, show an increase in freezing tolerance in response to low non-freezing temperatures, a phenomenon known as cold acclimation (Miura and Furumoto 2013). By contrast, some important crops such as rice, maize, tomato and cotton, originated from the tropical and subtropical climates, cannot to acclimatize to extreme temperatures and are therefore sensitive to chilling stress (Zhao *et al.* 2015).

When plants are exposed to any form of stress, the internal equilibrium dynamism rapidly changes, leading to excessive production of reactive oxygen species (ROS), which causes extensive damage to various cell structures. The response of plants to low temperature stress is a highly complex process involving multiple levels of regulation. When plants are exposed to cold stress, a series of physiological and biochemical alterations at the molecular and cellular levels are induced to increase plant cold tolerance and survival under cold stress conditions. These changes include increased accumulation of ROS-scavenging enzymes, rapid alterations in malondialdehyde (MDA) content, modifications in lipid compositions such as an increase in unsaturated fatty acid content which affects cell membrane fluidity, changes in protein and carbohydrate composition, accumulations of anti-freeze and anti-oxidative substances such as soluble sugar (SS), proline (PRO), superoxide dismutase (SOD), peroxidase (POD), catalase (CAT) among others (Hussain *et al.* 2016). After decades of intensive studies, we have begun to understand the cold-responsive (COR) system in plants at the transcriptional level (Wang *et al.* 2017). The C-repeat binding factor/dehydration-responsive element-binding factors (CBF/DREB) are critical transcription factors (TFs) which positively regulate the expression of downstream COR genes during cold acclimation. Also, CBF/DREB genes are activated by inducers of CBF expression (ICEs) through specific binding to the MYC recognition *cis*-elements in their promoter. The ICE-CBF-COR signal regulatory pathway has been identified as a critical COR transcriptional pathway during cold acclimation in many plants (Lee and Thomashow 2012). In *Arabidopsis*, there are three members of the *CBF*s (*CBF1/DREB1B*, *CBF2/DREB1C* and *CBF3/DREB1A*), encoding DNA-binding proteins of the AP2/ERF (APETALA2(AP2)/ethylene response factor) superfamily, which are rapidly induced in response to low temperature (Zhou *et al.* 2011). The *CBF4* gene, a novel homolog of CBF/DREB1 genes from *Arabidopsis*, was initially induced by abscisic acid (ABA) treatment under drought but not low temperature stress. Similarly, the *CBF4* gene is induced by cold stress in grape (Xiao *et al.* 2008). Moreover, CBF/DREB1 transcription activators are critical regulators of gene expression in the signal transduction of cold acclimation in *Arabidopsis* (Haake 2002). It is evident that the CBF cold response pathway has a major role in low temperature stress, especially during cold acclimation in temperate plants. But in the herbaceous monocot rice (*Oryza sativa*), which originated in the tropics, a novel MYBS3-dependent pathway has been identified as essential for cold tolerance. Molecular evidence indicated that the sequential expression of CBF and MYBS3 provided two complementary mechanisms for conferring cold tolerance, with the CBF-mediated process initiating the immediate cold shock response and the MYBS3-mediated system adjusting the long-term cold adaptation in rice (Su *et al.* 2010). Recently, a significant contribution of ROS-mediated gene regulation, rather than the CBF regulation, to a more vigorous transcriptional stress response in rice was revealed by RNA-Seq. The study revealed a ROS-dominated dynamic model underlying chilling environment adaptation and tolerance in rice (Zhang *et al.* 2016).

Cotton (*Gossypium* spp.), originally from tropical and subtropical climates, is the most important fibre crop for the textile industries and ranks highly among the edible crop oil producing crops in the world is sensitive to low temperature during its whole growth period, but is especially affected at the germination and seedling stages (Shan *et al.* 2007). The genus *Gossypium* includes 46 diploids (2n = 2x = 26) and 6 tetraploids (2n = 4x = 52) species, all the diploid cotton species subsequently diversified to produce eight groups, including groups A–G and K (Chakravarthy *et al.* 2012). All tetraploid cotton species originated through polyploidization between the A-genome and the D-genome species (Renny-Byfield *et al.* 2016). The A-genome species have experienced a long period of artificial selection for their high-quality spinnable fibre, whereas the D-genome species under natural selection maintained abundant genetic diversity and a number of desirable characters, such as fibre quality and resistance to salt, heat, drought, cold, insects and diseases (Kirungu *et al.* 2018). *Gossypium thurberi*, a wild diploid cotton species, is endemic to the desert sands of the southern part of Arizona in America and has adapted to the local temperature climate. The species is highly tolerant to low temperature and can survive low temperature as low as −6 °C (Liang *et al.* 1996). Researchers have identified some significant genes related to chilling tolerance in cotton, such as *GhDREB1* (Shan *et al.* 2007), *GhTIP1* (Li *et al.* 2009), *GhMT3a* (Xue *et al.* 2009), phospholipase Dα (Kargiotidou *et al.* 2010), *GbCBF1* (Guo *et al.* 2011), *GbPATP* (Liu *et al.* 2015) and *GhTPS11* (Wang *et al.* 2016). The overexpression of *DREB/CBF* genes has been found to confer cold tolerance to transgenic plants (Kang *et al.* 2013). Moreover, several *CBL* genes have been identified in cotton, through blast search of the CBL proteins from various dicotyledonous plants such as the reported cotyledons CBF sequences of *Arabidopsis* (Fowler *et al.* 2005), *Brassica napus* (Jing *et al.* 2008), soybean (Li *et al.* 2005; Kidokoro *et al.* 2015), among others plants in order to maximize the *CBF* genes discovery. In which 21 *CBF* genes were identified and classified into *GhCBF I*, *GhCBF II*, *GhCBF III* and *GhCBF IV* (Ma *et al.* 2014). These studies provided useful clues for understanding the mechanism of cold tolerance in cotton. However, due to the limited genomic sequences, these studies failed to provide a comprehensive interpretation of the transcriptomic changes in cotton in response to cold stress acclimation. To gain insight into the molecular networks underlying cotton chilling tolerance, more comprehensive genome-wide gene expression profiling studies are required.

In recent years, the whole-genome sequences of *G. raimondii* from D-genome and other three cultivated species such as *G. arboreum*, *G. hirsutum* and *G. barbadense* have been sequenced (Yuan *et al.* 2015). And therefore provides excellent genomic materials for better understanding the cold tolerance and transcriptome response mechanisms in cotton. In this study, a well-established whole-genome transcriptome analysis method based on RNA-Seq and incorporating real-time PCR was carried out to screen the differential gene expression in leaves of *G. thurberi* under cold treatment. We identified hundreds of COR genes and provided an overall overview of the regulatory network in response to cold stress in *G. thurberi*. These COR genes could be the potential candidate TFs for further functional validation and application in breeding for versatile and highly cold-tolerant cotton genotypes.

## Materials and Methods

### Plant material, growth conditions and cold treatments

Three cotton accessions were used in this research, *Gossypium thurberi* and two accessions derived from tetraploid cotton, *G. hirsutum*. *Gossypium thurberi* is a diploid cotton of the D-genome, it is known for its ability to tolerate cold stress (Kirungu *et al.* 2018), the other two are cultivated upland cotton, *G. hirsutum* cultivars, CRI50 and XLZ33, with differing ability to cold stress, CR150 is more tolerant to cold compared to ZLZ33. The seeds were obtained from the National Wild Cotton Nursery (Hainan, Sanya, China), maintained by the Institute of Cotton Research, Chinese Academy of Agricultural Sciences, China. The seeds were sterilized with 8 % sodium hypochlorite for 30 min and then washed with sterile water. Planting was carried out in germination boxes filled with sterilized sand, in a germination chamber, temperature set at 28 °C for 3 days to maximize germination. After germination, the seedlings were then transferred to small pots (bottom dimension 5 cm, top dimension 7 cm, depth 8 cm) containing well-watered mixtures of vermiculite and humus mixed in the ratio of 1:1. The seedlings were then moved to well-conditioned growth chambers, with 12-h light/dark and temperatures at 28 °C. For cold stress treatment, 3-week-old seedlings were moved to the cold treatment chamber, with conditions set at 12-h light/dark and 4 °C, the controlled seedlings were grown under normal condition. The leaf samples from cold stress treatment and controlled plants were collected at 0, 3, 6, 12, 24 and 48 h, and then stored under −80 °C awaiting RNA extraction and biochemical analysis. To minimize variations, 10 individuals’ plants were pooled into one independent biological replicate.

### Measurement of physiological and biochemical parameters in the three cotton species exposed to cold stress conditions

The relative electrolyte (ions) leakage from the cotton leaves was determined as described by Lu *et al.* (2018). Uniform sections of the leaves were obtained by the use of a punching machine with a diameter set at 1 cm, the leaf sections were then washed three times with sterilized deionized water. Twenty leaf segments were then placed in tubes containing 10 mL of sterilized deionized water and incubated at 25 °C. Four hours later, the electrical conductivity of the bathing solution (L1) was measured. The tubes were then autoclaved at 100 °C for 20 min, cooled to 25 °C before the final electrical conductivity (L2) was measured. The relative electrolyte/ion leakage was calculated by the formula = (L1 − L0)/(L2 − L0) × 100 (L0 = conductivity of deionized water).

Malondialdehyde levels were assessed by measuring thiobarbituric acid-reactive substances (TBARS). Superoxide dismutase, POD, PRO, soluble protein (SP) and SS were quantified as described by Mahmood *et al.* (2011). The concentrations of ABA, ethylene (ETH), gibberellic acid (GA) and indole acetic acid (IAA) in leaves were determined using the ELISA method using the following kits ABA (CK-E00005P, DG, Beijing, China), ETH (CK-E00021P, DG, Beijing, China), GA (CK-E00001P, DG, Beijing, China), IAA (CKE00015P, DG, Beijing, China), these four kinds of kits were utilized for the determination of each hormone contents. For the analysis 0.5 grams for each sample was used to determine concentration of the hormones through ELISA (Infinite M1000, TECAN, Switzerland). Three replicated experiments (two time technical repeats per biological replicate) were performed.

### RNA isolation, cDNA library preparation and RNA-Seq

Total RNA was isolated using the RNeasy Plant Mini Kit (Qiagen). The quantity and quality of RNA were checked by electrophoresis on 1 % agarose gels and NanoDrop 2000. RNA concentrations were measured using the Qubit RNA Assay Kit in Qubit 2.0 Fluorometer (Life Technologies, Carlsbad, CA, USA). The RNA integrity was assessed using the RNA Nano 6000 Assay Kit of the Agilent Bioanalyzer 2100 system (Agilent Technologies, Santa Clara, CA, USA). A total amount of 1 μg RNA per sample was used as input material for the RNA sample preparations. Sequencing libraries were generated using NEB Next Ultra TM RNA Library Prep Kit for Illumina (New England Biolabs Ltd, Beijing China) following the manufacturer’s recommendations and index codes were added to attribute sequences to each sample. The clustering of the index-coded samples was performed on a cBot Cluster Generation System using TruSeq PE Cluster Kit v4-cBot-HS (Illumina) according to the manufacturer’s instructions. After cluster generation, the library preparations were sequenced on an Illumina Hiseq 2500 platform and paired-end reads were generated.

### Bioinformatics analysis of RNA-Seq data

Raw data of fastq format were processed through in-house perl scripts to obtain clean data. The clean data were obtained by removing reads containing adapter ploy-N and low-quality reads from raw data. At the same time, the Q20 and GC content of the clean data were calculated. All the downstream analyses were based on clean data with high quality. Reference genome and gene model annotation files were downloaded from the genome website (http://mascotton.njau.edu.cn/html/Data) (Zhang *et al.* 2015). Index of the reference genome was built using Bowtie v2.0.6, and paired-end clean reads were aligned to the reference genome usingTophat2 (v2.0.9) software (Kim *et al.* 2013). Gene functions were annotated based on the following databases: Nr (NCBI non-redundant protein sequences), Nt (NCBI non-redundant nucleotide sequences), Pfam (Protein family), KOG/COG (Clusters of Orthologous Groups of proteins), Swiss-Prot (a manually annotated and reviewed protein sequence database), KEGG (Kyoto Encyclopedia of Genes and Genomes) orthology database (Kanehisa *et al.* 2008), GO (Gene Ontology). Gene Ontology enrichment analysis of the differentially expressed genes (DEGs) was implemented by the GOseq (Ashburner *et al.* 2000).

### Validation of RNA-Seq data by real-time RT-qPCR

A real-time RT-PCR (RT-qPCR) was performed. A total of 22 genes were selected from up-regulated, down-regulated and non-expressed genes (insignificantly expressed genes). The gene specific primers were designed by the use of primer 5 and *GrActin* gene forward sequence ‘ATCCTCCGTCTAGACCTTG’ and reverse sequence ‘TGTCCATCAGGCAACTCAT’ was used as an internal control **[see****Supporting Information—Table S1****]**. The RT-qPCR was performed using a Bio-Rad CFX96 Real-Time instrument and the LightCycler FastStart DNA Master SYBR Green I kit (Roche, Basel, Switzerland) on a new set of three replicates for each sample.

### Functional characterization of the candidate genes through virus-induced gene silencing in *G. thurberi*

The TRV2 (tobacco rattle virus) vectors were prepared and introduced into *Agrobacterium tumefaciens* strain GV4104 (Gao *et al.* 2011). To monitor the silencing efficiency, the TRV2-PDS vector was constructed as a visual marker. Primers used to generate TRV vector, for the *CBF4* gene forward sequence ‘GTGAGTAAGGTTACC GAATTCGGTTGATTCTGGGTCGGTTTC’ and the reverse sequence ‘CGTGAGC TCGGTACCGGATCCGACTTCTTATTA’ and the second gene, *ICE2* forward sequence ‘GTGAGTAAGGTTACCGAATTCTGGTTATCAGGTGGAGG’ and the reverse sequence ‘CGTGAGCTCGGTACCGGATCCAATGGAAAT’, were inserted into the vector pTRV2 of tobacco rattle virus (TRV) via EcoRI and BamHI digestion sites to construct a 35S promoter-driven pTRV2:*CBF4* and pTRV2:*ICE2*, respectively. The recombinant vector was transformed into the competent cells of *A. tumefaciens* LBA4404 by freeze-thaw method, and the bacterium LBA4404 containing pTRV1 vector was used as auxiliary bacteria, being pTRV1 can replicate and move systemically without RNA2, mainly constructed from a T-DNA vector containing duplicated CaMV 35S promoter, nopaline synthase (NOSt) terminator and cDNA clone of TRV RNA1 of Ppk20 strain (Liu *et al.* 2002). The cotyledons of cotton seedlings were infected by injection, and pTRV:PDS (phytoene desaturase) was used as a positive control. Plants without infection and empty vector pTRV:00 were used as negative control. The *Agrobacterium* culture was agroinfiltrated into the two expanded cotyledons of 10-day-old soil-grown seedling of *G. thurberi*. The cotton seedlings were then grown in a greenhouse condition, with temperature and light cycles set at 28 °C, and 16-h light/8-h dark cycle, respectively. At least 24 seedlings were inoculated for each construct with three replications, with eight plants per replication. At 14 days after *Agrobacterium* inoculation, and when virus-induced gene silencing (VIGS) was established in >75 % of the seedlings 14 days after *Agrobacterium* inoculation, as determined by the whitening/albino nature of the plant leaves. The silenced seedlings were subjected to cold stress treatment; this was done at the three true leaf stages. Cold stress was imposed on the wild types, positive control with empty vector and the gene silenced seedlings, by transferring them to a cold chamber with the temperature set at 4 °C for 3 days. After 3 days of cold stress treatment, the leaf samples were collected and MDA, PRO and SOD assayed.

### Statistical analysis

The plant’s responses to cold stress were determined through morphological, physiological, biochemical and transcriptome analysis. A complete randomized block design (CRBD) was used. All the results presented are given as means with standard errors. All analyses were undertaken using IBM SPSS version 25 (IBM SPSS 2017). Normality and homogeneity of variance were analysed by Kolmogorov–Smirnov and Levene’s tests. The significance of the results was assessed using independent samples *t*-test or two-way ANOVA.

## Results

### Phenotypic responses to cold stress

**Figure 1.**
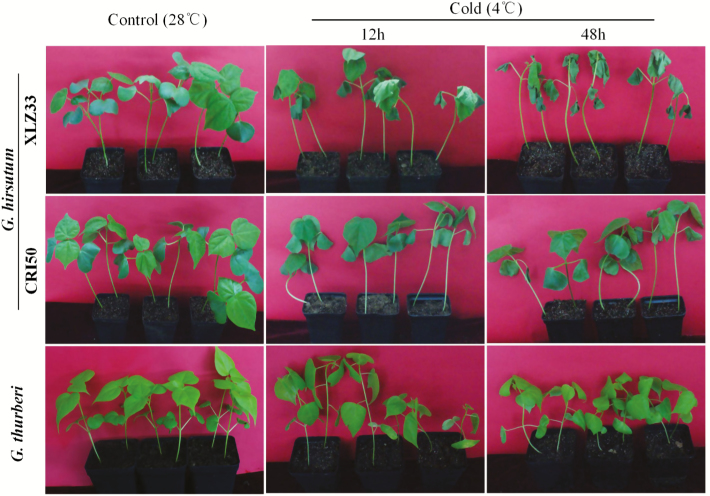
Phenotypes of 3-week seedlings of *G. thurberi* and two *G. hirsutum* accessions under cold stress (4 ℃) for 48 h. Seedlings grown under normal conditions (28 °C) were used as controls. *Gossypium hirsutum*-CRI50 is cold-tolerant accession and *G. hirsutum*-XLZ33 is cold-sensitive accession.

To accurately evaluate the tolerance of *G. thurberi*, we compared the phenotypic changes of *G. thurberi* versus two accessions of *G. hirsutum*, CRI50 and XLZ33, which are tolerant and sensitive to cold stress, respectively. *Gossypium thurberi* is the northernmost species of *Gossypium*, reaching southern Arizona. Since it occurs at elevations as high as 2000 m, it is growing in a temperate zone climate, the only *Gossypium* to do so. It achieves this adaptation by undergoing leaf senescence and abscission (simultaneously with fruit maturation) in the autumn and becoming fully dormant by the winter months. In this case, the move towards dormancy is not triggered by declining moisture supplies in the soil, but rather by the advancing season (day length, temperature regime or some combination of factors) (Walker and Natwick 2006). A significant phenotypic difference was observed in XLZ33, its leaves wilted after 12 h of cold treatment, while CRI50 and *G. thurberi* leaves were not affected. Moreover, after 48 h of cold stress treatment, ZLZ33 were severely necrotic and wilted, while *G. hirsutum* CRI50 and *G. thurberi* leaves only showed symptoms of wilting but were not necrotic (Fig. 1). After 48 h of cold stress treatment, a high number of ZLZ33 seedlings died, while *G. thurberi* and CR150 seedlings achieved 100 % recovery. These observations suggested that *G. thurberi* had a higher ability to tolerate cold stress compared to the tetraploid cotton, *G. hirsutum*. The results obtained correlated positively with previous findings, which showed that *G. thurberi* has a higher ability to tolerate cold stress (Walker and Natwick 2006).

### Physiological and biochemical analysis in the leaf tissues of *G. thurberi* and two other upland cotton under cold stress condition

**Figure 2.**
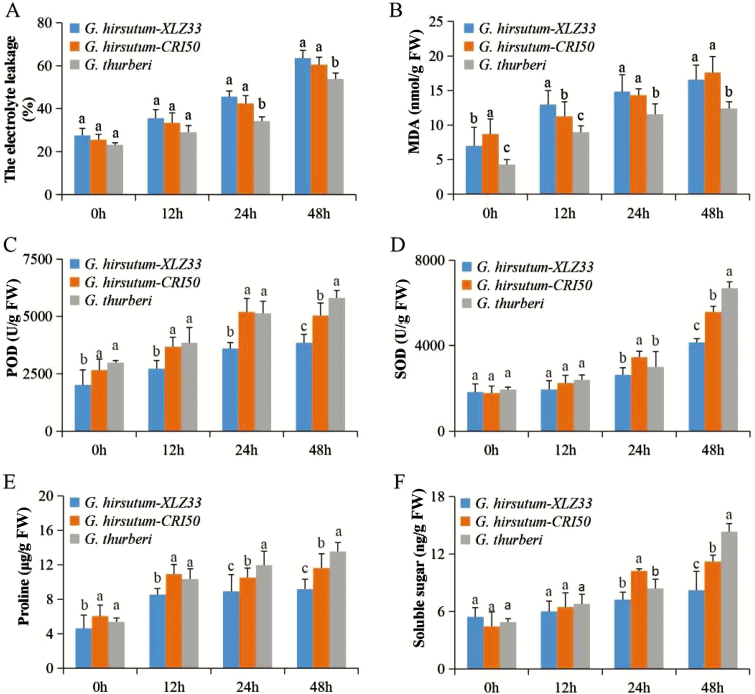
Physiological analysis of *G. thurberi* in response to cold stress. (A–F) The relative electrolyte leakage, the concentrations of MDA, POD, SOD, PRO and SS were determined for *G. thurberi* at 4 °C for 0, 12, 24 and 48 h. Three biological replicates were used. The different letters labelled above columns indicate a significant difference at *P* ≤ 0.05 level. The columns with the same letters mean no significant difference (*P* > 0.05) between different cotton genotypes. Bar indicates standard error.

Variations of the physiological and biochemical indices *thurberi* among the three cotton species, *G. thurberi*, ZLZ33 and CRI50 seedlings, were investigated after cold stress treatment. The cell membrane stability is a significant trait in determining the tolerance level of a plant to various abiotic stress factors (Mansour 2013). In evaluating the cell membrane stability through relative electrolyte leakage, the electrolyte leakage increased from 23.2 to 55.8 % after 48 h of cold stress exposure (Fig. 2A). Furthermore, the MDA content increased over time, but the increasing tendency slowed down after 12 h of cold treatment in *G. thurberi* but the MDA levels in the two upland cotton, CRI50 and XZL33, were higher, though relatively low in CRI50 compared to XZL33 at time 0 h, lower at 12 h and not statistically different at 24 and 48 h (Fig. 2B), an indication that the two upland cotton accession of the upland cotton species had a similar capacity to tolerate the effect of cold stress. These observations suggested an element of acclimation to cold stress by *G. thurberi* seedlings after 12 h of cold exposure, which enabled them to protect their membrane stability as evidenced by the low level of electrolyte leakage. Moreover, *G. thurberi* exhibited significantly increased levels of various antioxidant enzymes analysed, such as POD, SOD, PRO and SS after cold treatment at 4 °C compared to the other two upland cotton species, the POD and SOD concentration levels between *G. thurberi* and the two upland cotton accessions varied but were not statistically different at 0, 12 and 24 h, more so between *G. thurberi* and *G. hirsutum*-CRI50, but as the time of stress exposure increased, significant differences were observed among the three cotton species at 48 h of cold stress exposure, and the three cotton species showed significant differences with *G. thurberi* registering the highest levels of both POD and SOD antioxidant enzymes concentrations (Fig. 2C and D). While in evaluating the PRO content among the three cotton species, CRI50 and *G. thurberi* showed no significant differences at 0 and 12 h; however, at 24 and 48 h of cold stress exposure, the three cotton species exhibited significant differences in the level of PRO content, with *G. thurberi* registering the highest concentration levels (Fig. 2E). About the SS, no significant differences were observed at 0 and 12 h, but variations were observed at 24 and 48 h, with high levels of SS in the leaves of *G. thurberi* compared to the two upland cotton accessions, CR50 and XLZ33 (Fig. 2F). The increased concentration levels of the antioxidant and SS in the leaf of *G. thurberi* showed that *G. thurberi* had the ability to tolerate very low temperatures compared to the other two upland cotton species, when plants are exposed to cold or chilling conditions of temperatures between 0 and 15 °C, hydrogen peroxide (H_2_O_2_) may accumulate (Kocsy *et al.* 2001). Thus, the increased levels of antioxidant enzymes could be playing an important role in protecting the plant cells from ROS damage (Caverzan *et al.* 2016). The results are in agreement with previous findings in which various antioxidant enzymes are more in the leaves of stress-tolerant genotypes compared to susceptible varieties when they are exposed to either abiotic or biotic stress conditions (Sairam *et al.* 1997). Moreover, knockdown of two novel *CYP450* genes in upland cotton increased drought and salt stress susceptibility as evident by increased levels of oxidant and significant decline in antioxidant enzymes in the leaves of the VIGS plants (Magwanga *et al.* 2019a). Reactive oxygen species are oxygen-containing substances of metabolites and their derivatives generated through oxidation in plants, directly or indirectly. Reactive oxygen species play a vital role in stress signal transduction, but excessive active oxygen oxidation harms the plant (Gill and Tuteja 2010). Therefore, plants also produce a series of active oxygen-scavenging enzymes, for example, POD, CAT, SOD among others.

### Plant phytochemical assays on the tissues of wild cotton, *G. thurberi* under cold stress conditions

**Figure 3.**
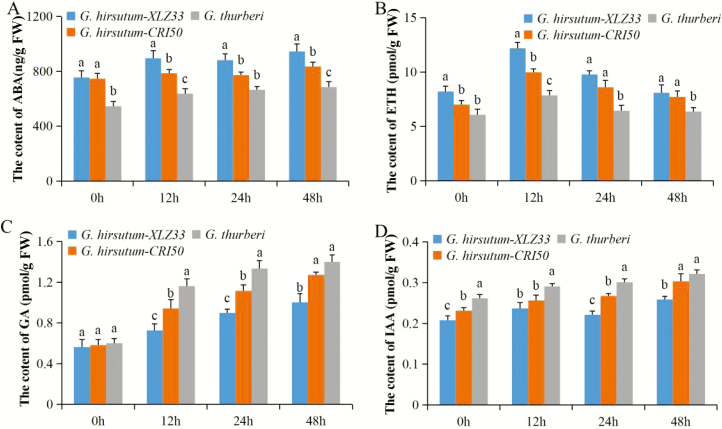
Phytohormones analysis in the leaf tissues of *G. thurberi* under cold stress. (A) Quantification of ABA, (B) quantification of ETH, (C) quantification of GA and (D) quantification of IAA. Three biological replicates were used. The different letters (a/b) labelled above columns indicate a significant difference at *P* ≤ 0.05 levels. The columns with the same letters mean no significant difference (*P* > 0.05) between different cotton genotypes. Bar indicates standard error.

In elucidating the inherent traits in *G. thurberi* which enables them to be more tolerant to cold stress, we analysed various plant phytohormones, such as gibberellic hormones (GA), ABA, ETH and IAA. The ability of the plants to regulate the phytohormones productions, distribution and or signal transmission, do aids their ability to coordinate and regulate growth and in turn increases their level of tolerance to various forms of abiotic stress factors (Colebrook *et al.* 2014). Ethylene hormone (ETH) at 0 h was relatively low across the three cotton species; however, with the increase in stress exposure, the evolution of ETH hormone increased rapidly hitting the pick after 24 h of cold stress exposure then showed a gradual decline at 48 h (Fig. 3A). A similar observation was made in grapevine in which ETH concentration increased and normalized with an increase in cold stress exposure (Sun *et al.* 2016). Abscisic acid in relation to GA and/or IAA work antagonistically, increase in ABA leads to a significant decline in either GA or IAA (Peleg and Blumwald 2011). In the evaluation of the three antagonistic plant hormones, ABA, GA and IAA, ABA showed a gradual increase across the three cotton species; though lower than the concentration levels of GA and IAA (Fig. 3B–D). The results obtained were in agreement with previous findings in which various hormones such as GA have been found to playing a significant role in enhancing cold stress tolerance in plants (Colebrook *et al.* 2014). Moreover, emission of ETH plants at low concentration levels is an adaptive mechanism for plants to avoid the lethal damage by the environmental stressors; furthermore, ETH also do regulates other diverse activities such as seed germination, growth, formation of apical hook, to organ senescence, fruit ripening, abscission and stress responses (Pei *et al.* 2017). The concentration levels of GA and IAA were varied, even though the two hormones are known to promote plant growth and development (Tanimoto 2005), the level of IAA was lower than GA in the three cotton species under cold stress, the results are in agreement with previous findings in which found that cold stress inhibits the root gravity response in *Arabidopsis* by 50 %, a process mainly regulated by the IAA (Shibasaki *et al.* 2009), the results, therefore, showed that cold decreases the internal IAA levels in plants.

### Transcription analysis of *G. thurberi*

To gain insight into the gene expression pattern in the leaf tissue of *G. thurberi* under cold condition, 10 cDNA libraries were constructed under normal (control) and low temperature (cold treatment) at 0, 6 and 12 h, respectively. The samples were coded as follows CK0-1, CK6-1, CK12-1, CK0-2, CK6-2, CK12-2, CS6-1, CS12-1, CS6-2 and CS12-2. The cDNA libraries were sequenced by Illumina HiSeq2500 platform using the paired and end method. After filtering out low-quality reads, raw reads containing ‘N’ and adaptor sequence, 34.48–43.66 M clean reads were collected. The clean reads were aligned to the diploid D-genome cotton reference genome (*G. raimondii*), whose progenitor is the putative contributor of the Dt-sub genome of the tetraploid-cultivated cotton species. Approximately 75.53–78.25 % clean reads were mapped to the reference genome and multiple mapped clean reads in each library were excluded from further analysis. Finally, a total of 22.91–30.83 M uniquely mapped reads were used for subsequent analysis (Table 1). The mapped sequences were assembled with Cufflinks software inference to the *G. raimondii* genome (https://www.cottongen.org/). The RNA-Seq assays revealed that there were a total of 35 617 unigenes of which 97.52 % (34 733 unigenes) were annotated genes and 2.48 % (884 unigenes) novel genes based on this analysis **[see****Supporting Information—Table S2****]**.

**Table 1.** Summary of the sequencing data of the *G. thurberi* transcriptome. CK and CS: normal temperature control and cold stress treatment, respectively. Units: M, million.

Sample	Mapped read (M)	Mapped ratio (%)	% ≥ Q30
CK0-1	34.17	78.25	91.59
CK6-1	33.1	75.88	91.33
CK12-1	26.04	75.53	92.16
CS6-1	32.39	77.33	92.03
CS12-1	26.9	76.91	92.02
CK0-2	40.02	67.53	93.84
CK6-2	41.05	69.79	96.03
CK12-2	48.56	69.50	96.32
CS6-2	30.08	68.09	95.42
CS12-2	43.78	72.48	96.35

**Figure 4.**
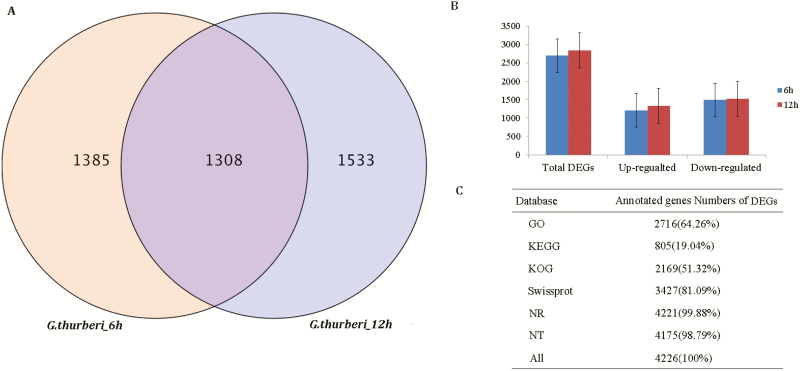
Summary of DEGs in *G. thurberi*. (A) Venn diagram of DEGs identified in *G. thurberi* in response to cold stress. The purple zone meant common DEGs at 6 and 12 h post to cold stress. (B) The summary of DEGs was shown in bar graph. The blue bars represent the DEGs at the 6 h of cold stress treatment. The red bars represent the DEGs at the 12 h post-cold stress treatment. Bar indicates standard error. (C) DEGs annotation of *G. thurberi* by different databases.

To identify the DEGs in *G. thurberi* under cold stress and normal conditions, the transcript abundance of each of the genes was estimated by fragments per kilobase exon per million fragments mapped (FPKM). DESeq and the *Q*-values were employed and used to evaluate the differential gene expression pattern. A cut-off *P*-value < 0.01 adjusted by false discovery rate (FDR) and fold change ≥ 2 was used to identify DEGs. A total of 4226 genes showed differential expression after 6 and 12 h of cold stress treatment (CK_6h vs. CS_6h, CK_12h vs. CS_12h). The distribution of the DEGs revealed that 2693 and 2841 genes were expressed in the leaves at 6 and 12 h of cold stress treatment, respectively, and that 1308 DEGs were identified at both time points (Fig. 4A). Among these DEGs, 1205 and 1322 up-regulated genes, 1488 and 1519 down-regulated genes detected under cold stress at 6 and 12 h time points, respectively; however, the number of up-regulated genes at 12 h of stress exposure were more than the up-regulated genes at 6 h of cold stress exposure (Fig. 4B). The results showed that more genes were down-regulated in the leaves in response to cold stress. Interestingly, compared with the number of DEGs at 6 h post-stress exposure/treatment, there were more DEGs at 12 h of post-stress treatment, which indicated that more COR genes were induced by an increase in exposure. Moreover, analysis of the DEGs through various databases revealed higher percentage values, for instance, the DEGS with GO terms were 2716 (64.26 %), KEGG values were detected for 805 genes accounting for 19.04 % (Fig. 4C). The detection of various functions through the GO, KEGG, KOG among others indicated that these genes have a putative role in enhancing cold stress tolerance in *G. thurberi*.

### Functional analysis of the DEGs under cold stress condition

**Figure 5.**
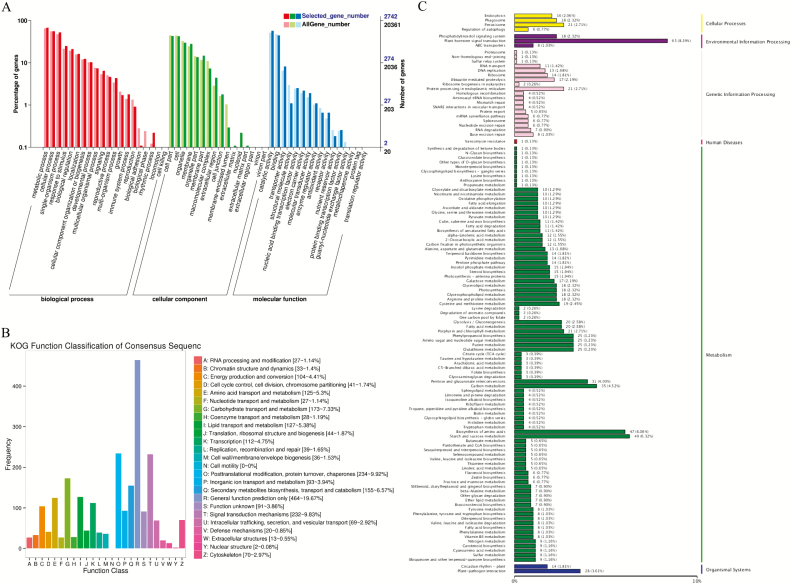
GO, KOG, KEGG enrichment of DEGs. (A) GO enrichment of DEGs; (B) KOG enrichment of DEGs; (C) KEGG enrichment of DEGs.

Gene Ontology enrichment analysis showed that a total of 2716 (64.26 %) DEGs in *G. thurberi* were annotated, and were involved in all the GO functional annotations, biological processes (BP), molecular function (MF) and cellular component (CC). For the BP, metabolic process, cellular process, single-organism process and response to stimulus were the top terms, which implied that a high degree of metabolic activity changes occurred in response to cold stress. For the category of CC, cell part, cell, organelle and membrane were enriched, which suggested that the cell membrane and cellular homeostasis played an important role in the process of cotton acclimation to cold stress conditions. The most abundant terms for MF were the catalytic activity, followed by binding and ‘transporter activity, which revealed that complex enzymes system and other biological catalytic processes were involved in response to cold stress. KOG enrichment analysis showed that many of DEGs were most significantly enriched in posttranslational modification, ‘protein turnover, chaperones formations, signal transduction mechanisms, secondary metabolites biosynthesis, ‘transport and catabolism of the orthologous proteins under abiotic stress condition (Fig. 5A). To gain further understanding of the biological molecular mechanism and regulatory network of DEGs in *G. thurberi* in relation to cold tolerance, KEGG enrichment pathway analysis was performed. A total of 805 (19.04 %) DEGs in *G. thurberi* were annotated to KEGG pathways. Among these pathways, the plant hormone signal transduction (ko04075) pathway was the most significant enrichment. A total of 65 genes involved in this pathway were affected by low temperature. Moreover, 265 DEGs were annotated as different types of TFs, out of which 225 TFs were obtained. Among these TFs, 138 genes were up-regulated, while the rest of the genes were down-regulated after cold treatment in *G. thurberi* (Fig. 5B; **see****Supporting Information—Fig. S1**). According to functional annotation, these TFs were classified into numbers of categories, such as *ERF* (ethylene response TFs) family (34 TFs), *bHLH* family (32 TFs), WRKY family (25 TFs), MYB family (17 TFs), TCP (9 TFs) and NAC family (9 TFs). The TFs ERF (34 genes, 15.1 %), bHLH (32 genes, 14.2 %), WRKY (25 genes, 11.1 %) and MYB (17 genes, 7.5 %) were the top four major families of the cold-regulated TFs. In addition, we observed that the expression of *CBF1*, *CBF2*, *CBF3/DREB1A* and *DREB2A*, which bind to the C-repeat or dehydration response element promoter’s genes, were significantly altered after cold treatment, suggesting that these TFs family might be playing an important regulatory role in responses to cold stress in *G. thurberi*. This result was consistent with previous studies that demonstrated that plant hormones play crucial roles in a diverse set of developmental processes as well as in the response to biotic and abiotic stress (Verma *et al.* 2016).

Signal transduction has been observed in response to abiotic and biotic stress in many plant species (Czarnocka and Karpiński 2018). A total of 232 DEGs were identified related to different signal transduction mechanisms. Calcium (Ca^2+^), a universal second messenger in cellular signal transduction, plays a vital role in the plant cold stress response. The cytosolic-free concentration changes within seconds in plants when subjected to cold stress, followed by several signals mediated by a series of protein phosphorylation cascades (Luan 2009). In our data, large numbers of the DEGs involved in signal transduction were indeed related to the calcium-dependent signal pathway, such as CDPKs (calcium-dependent protein kinases), *CIPKs* (CBL-interacting protein kinases) and *CMLs* (calmodulin-like protein). The majority of the genes from these three gene families from *G. thurberi* were up-regulated in response to cold.

Carbohydrate transport and metabolism play a core role in plant response to cold stress. All of the 176 DEGs in carbohydrate transport and metabolism were identified under cold stress in *G. thurberi*. Among these DEGs, 19 genes were up-regulated in all stages of *G. thurberi* and 35 genes were down-regulated at 6 and 12 h of cold stress exposure. Aquaporins are transmembrane proteins, which form channels in intracellular and plasma membranes to facilitate the rapid movement of water and play a crucial role in plant water relations. Plant aquaporins are classified into five main subfamilies including the plasma membrane intrinsic proteins (PIPs), tonoplast intrinsic proteins (TIPs), nodulin-26-like intrinsic proteins (NIPs), small basic proteins (SIPs) and X intrinsic proteins (XIPs) (Kapilan *et al.* 2018). In the present study, 16 *PIPs*, three *TIPs*, two *NIPs* and one *SIP* genes were induced by cold stress. Moreover, in *Arabidopsis*, most of the *PIP* genes were down-regulated by cold stress, except for the *PIP2;5* which was up-regulated during cold exposure. Furthermore, overexpression of the *PIP2;5* gene in *Arabidopsis* have been found to play a role in alleviating the effects of low temperature on plant cell hydraulic conductivity and growth (Lee *et al.* 2012). Ten out of 16 *PIPs* genes from *G. thurberi* were significantly up-regulated under cold stress treatments, especially gene6326 (annotated as *PIP2-2*) exhibited 4-fold higher expression compared to the control during cold stress. In previous studies, a member of tonoplast intrinsic protein *GhTIP1:1* was responsive to cold stress and contributed to freezing tolerance in upland cotton. Our transcriptome data analysis showed three differential expressed *TIPs* genes (including gene8287, gene8689 and gene31507); one *NIPs* gene (gene10377) and one *SIPs* gene (37783) were highly up-regulated under cold stress (Fig. 5C; **see****Supporting Information—Table S3**). All results suggested that aquaporins may play a key role in plant response to cold stress by maintaining the uptake and movement of water in the plant body.

### Validation of RNA-Seq results by quantitative real-time PCR

**Figure 6.**
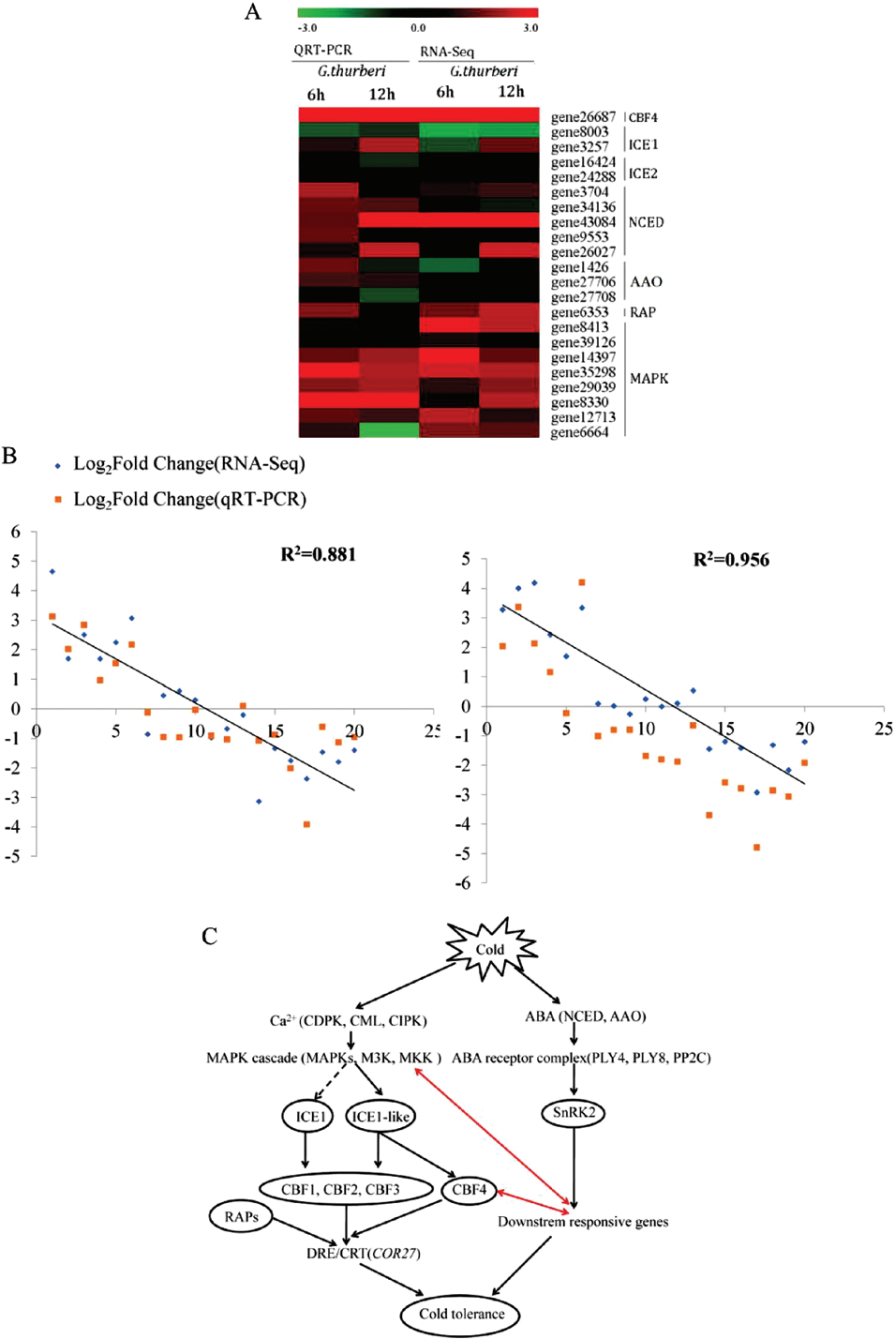
RT-qPCR validations of transcript levels evaluated by RNA-Seq in *G. thurberi* under cold stress for 6 and 12 h. (A) Heat map depicting log2 (fold change) of transcription factor; (B) regression analysis of the log2 (fold change) of RNA and qRT-PCR expression analysis of the DEGs; (C) a predicted model of cold response transcriptional network in *G. thurberi*. Red lines indicated the crosstalks between different signalling pathways predicted in this analysis. Y-axis—relative RNA-Seq expression levels and X-axis—relative RT-qPCR expression levels

Seven different gene families were analysed, *CBF4*, *ICE1*, *ICE2*, *NCED*, *AAO*, *RAP* and *MAPK* gene families were profiled. The members of *CBF4*, *MAPK* and *RAP* genes were highly up-regulated compared to other families (Fig. 6A). Through correlation analysis, RT-qPCR results were significantly correlated to the RNA-Seq data both at 6 and 12 h points under cold stress (*R*^2^ = 0.881 and 0.956, respectively) in *G. thurberi* (Fig. 6B). Moreover, in the schematic representation of the possible genes, *ICE1*, *ICE1-like* and the *CBF4* were more integral in enhancing cold stress tolerance in *G. thurberi* (Fig. 6C).

### Knockdown of *CBF4* and *ICE2* genes in cotton through VIGS seedlings compromises their ability to tolerate cold stress

**Figure 7.**
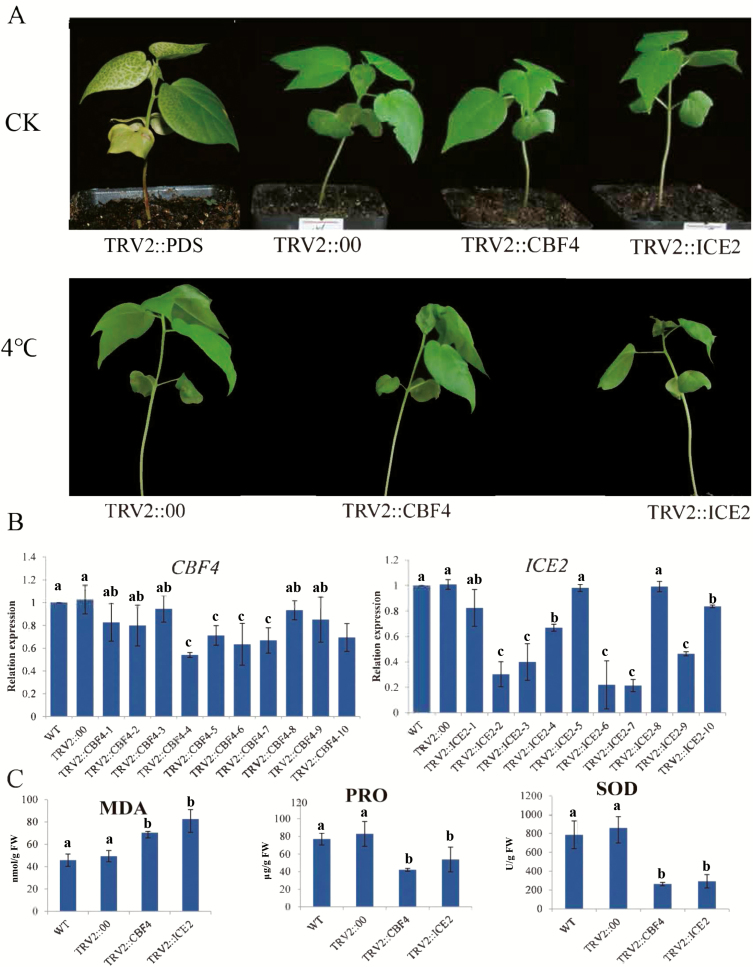
Virus-induced gene silencing (VIGS), RT-qPCR analysis and biochemical evaluation of the VIGS plants. (A) No obvious symptoms in the leaves of the TRV:00-infected plant (positive control); in each experiment, six plants were used and there were three replicates. (B) RT-qPCR analysis of the changes in the expression level of the two *CBL4* and *ICE2* genes in the leaf tissues of cotton plants treated with VIGS under cold stress conditions. (C) Quantitative determination of MDA, PRO and SOD in leaves of the wild type (WT), control and the silenced VIGS plants, in *CBF4* and *ICE2* silenced plants; line CBF4-4 and ICE2-7 were used, respectively. All measurements were done after 8 days of stress exposure. In (B) and (C), each experiment was repeated three times. Error bars of the biochemical trait measurements represent the standard deviation of three biological replicates. Different letters (a/b) indicate significant differences between WT and VIGS plants (two-tailed; *P* < 0.01).

To further investigate the functions of candidate genes, *CBF4* and *ICE2*, the VIGS *TRV-GhPDS*, *TRV-Ctrl*, *TRV-CBF4* and *TRV-ICE2* plants were observed under cold stress. Albino leaves were observed in *TRV-PDS*-inoculated seedlings after 7 days of inoculation. Compared with infected seedlings, we found that control seedlings were rapid growth after 20 days of inoculation. And, the difference was not observed between infected seedlings; moreover, cold stress was imposed after three leaf stage, the VIGS plant’s ability to tolerate cold stress was highly affected, the leaves wilted after cold stress treatment (Fig. 7A). Moreover, the expression levels of *CBF4* and *ICE2* were checked by RT-qPCR, their expression levels were down-regulated compared to their expression in the leaves of the *TRV-Ctrl* seedlings, line CBF4-4 and line ICE2-7 were used for further evaluation, being the *CBF4* and *ICE2* genes expression were significantly down-regulated (Fig. 7B). Finally, MDA, PRO and SOD were assayed on the leaves of VIGS plants and their wild types under cold stress conditions. The VIGS plants exhibited a higher concentration level of MDA and a significant reduction in the levels of PRO and SOD compared to the wild types (Fig. 7C). The results obtained were in agreement with the previous findings in which knockdown of a regulatory gene affects the normal growth and development of the plants being the plant’s ability to tolerate the stress of target become highly compromised (Jahan *et al.* 2015). Moreover, knockdown of CYP450 genes significantly reduced the tolerance level of upland cotton, *G. hirsutum* to salt and drought stress factors (Magwanga *et al.* 2019b). Furthermore, RNAi of a stress-responsive gene increases susceptibility of the plant to environmental stresses, for instance, overexpression of a novel trihelix transcription factor enhanced drought and salt stress tolerance in transgenic *Arabidopsis*, but when the same gene was knockdown, the cotton VIGS plants ability to tolerate the two forms of stresses was affected significantly, as was evident by high concentration levels of oxidant enzymes and significant reduction in the levels of various antioxidant enzymes (Magwanga *et al.* 2019b).

## Discussion

Cold stress limits cotton growth and development, its effect has caused massive losses in the cotton production industry (Holaday *et al.* 2016). Cotton originated from the tropical and subtropical climates and therefore lacks the cold acclimation mechanism and is sensitive to low temperature throughout the growth period (Lee and Fang 2015). Particularly, if exposed to low temperature in the period of germination or seedling, cotton growth and development would be slow and at the time of budding, flowering and maturity would be delayed (Gai *et al.* 2008). In addition, low temperature not only affects the yield of cotton but also the quality of fibre (Liu *et al.* 2015).

Even though plant response to cold stress is a highly complex mechanism, after decades of intensive studies we have begun to understand the COR system in plants (Wang *et al.* 2017). However, the cold tolerance mechanism in cotton has gained minimal attention so far. In this study, we aimed at unravelling the cold stress-responsive mechanism in two cotton, *G. thurberi* and two accessions of tetraploid cotton, *G. hirsutum*. The results showed that after 48 h of cold stress at 4 °C, morphological and physiological traits of the diploid wild cotton *G. thurberi* underwent drastic changes to acclimatize to the cold stress condition. The changes, therefore, showed that *G. thurberi* was highly tolerance to cold stress, the results were consistent with previous studies (Liang *et al.* 1996). The SS and PRO are the most effective organic osmolytes in plants, which can help stabilize proteins and cell structures under stressful environmental conditions (Naser *et al.* 2010). These compounds can also act as free-radical scavengers, protecting against oxidation by removing excess ROS, and also re-establishing the cellular redox balance (Thalmann *et al.* 2016). There is a positive correlation between SS and PRO accumulations, and plant stress tolerance (Dikilitas *et al.* 2017). In this study, 49 and 83 genes were involved in ‘starch and sucrose metabolism (ko00500)’ and ‘arginine and proline metabolism (ko00330)’ pathways, respectively, and were regulated by cold stress. Half of these DEGs were highly up-regulated, which suggested that these cold-induced genes might promote the accumulation of SS and PRO in *G. thurberi* to enhance cold tolerance. The results obtained by the tissue analysis of *G. thurberi* under cold stress condition showed a marked increase in PRO and SS. The increased levels of PRO and SS indicated that they are the major osmolytes responsible for osmotic adjustment in cotton under low temperature condition. Moreover, the plant endogenous hormones had significant changes under cold stress.

To gain insight into the morphological, physiological and hormonal changes in *G. thurberi*, we performed transcriptome sequencing analysis under cold stress condition. A total of 35 617 genes were obtained, in which 4227 genes were differentially expressed under cold stress in *G. thurberi.* High numbers of DEGs were significantly enriched in various processes such as single-organism process, metabolic process, response to the stimulus, membrane, organelle part, membrane part, catalytic activity and nucleic acid binding transcription factor, as obtained through GO and KEGG enrichment analysis. Moreover, KEGG enrichment analysis proved that many of the genes were involved in plant hormone signal transduction, ‘starch and sucrose metabolism. These results of KEGG enrichment analysis demonstrated signal transduction, the sugar transport and metabolism played an important role in response to cold stress. Furthermore, ROS are oxygen-containing substances of metabolites and their derivatives generated through oxidation in plants, directly or indirectly. Reactive oxygen species play a vital role in stress signal transduction, but excessive active oxygen oxidation harms the plant (Gill and Tuteja 2010). Therefore, plants also produce a series of active oxygen-scavenging enzymes, for example, POD, CAT, SOD among others. In this study, the expression of *RBOH* (respiratory burst oxidase homolog) gene, which is responsible for the accumulation of H_2_O_2_, was rapidly up-regulated in leaf tissues at 6 h of cold treatment. Simultaneously, ROS-scavenging systems were markedly inducted, showing different expression patterns under cold stress. In total, 16 POD genes, 9 SOD genes and 1 CAT gene were found, and most of them were up-regulated. Interestingly, the CAT gene (gene6557) showed no expression at 6 h, but was significantly up-regulated at 12 h of cold stress treatment. Moreover, plant hormones such as auxin, cytokinins, gibberellin (GA), ABA, ETH, brassinosteroids (BR), jasmonic acid (JA) and salicylic acid (SA) are small molecules that play significant roles throughout the life span of plants, especially when plants encounter adversity (Verma *et al.* 2016). In our research, we found a series of DEGs were annotated plant hormone signal transduction (ko04075) and were involved in series of signalling pathways, for instance, 36 DEGs were involved in auxin-mediated signalling pathway (GO:0060774), 10 DEGs in ABA-activated signalling pathway (GO:0009738), five DEGs in GA-mediated signalling pathway (GO:0009740), five DEGs in ETH-activated signalling pathway (GO:0009873) and finally eight DEGs were involved in JA-mediated signalling pathway (GO:0009867). In the present study, most of these DEGs related to plant hormone signal transduction were up-regulated in all stages, combined with the changes in endogenous hormones concentrations in cotton seedling under cold treatment, the results suggested that plant hormone signal transduction plays a core role in response to cold stress.

Plants have a complex molecular mechanism network that enables them to respond to cold stress. Generally, the cold stress signal is first received by the receptors on the plant cell membrane, which results in the generation of many secondary messengers, such as Ca^2+^ ions cascades, hormones and ROS (Miura and Furumoto 2013; Zhang *et al.* 2017). The signal molecules transmit the stress signals into the nucleus, which then leads to the activation of related genes to induce responses to cold stress (Chinnusamy *et al.* 2007). Transcription factors are fundamental to the regulation of cellular pathways in response to adverse stresses in many plants. In this study, we identified several types of differentially expressed TFs in response to cold stress in *G. thurberi*, including *ERF*, *TCP*, *WRKY*, NAC, *bHLH*, *MYB* family and among others. The effects of ETH on plants under cold stress have been elucidated (Wang *et al.* 1990; Abiri *et al.* 2017). The *ERF* TFs are implicated in many diverse functions in cellular processes, such as hormonal signal transduction, response to biotic and abiotic stresses, regulation of metabolism and in developmental processes in various plant species (Rehman and Mahmood 2015). In this study, we found >12 % of the *ERF* TFs to be differentially expressed under cold stress. It has been reported that overexpression of transcription factor *TERE2/LeERE2* in tobacco and tomato could enhance cold tolerance by facilitating ETH biosynthesis, which supports the important role of *ERF* in plant cold tolerance (Zhang and Huang 2010). In addition, dehydration-responsive element-binding (*DREB*) protein, which belongs to the subfamily of AP2/EREBP TFs, specifically binds to the promoter regions of downstream genes, activates or suppresses the transcription of these genes and finally enhance plant stress tolerance (Kizis *et al.* 2001). The previous study has reported that a cotton *DREB* gene (*GhDREB*) conferred enhanced tolerance to drought, salinity and freezing stresses in transgenic wheat (Gao *et al.* 2009). The same gene was significantly induced after cold treatment in *G. thurberi*, suggesting that this gene may be critical in response to cold stress in cotton. In addition, the *TCP* family is a group of plant-specific TFs that plays an important role in the growth and development of plants (Chai *et al.* 2017). In this study, >14 % of the *TCP* TFs were differently expressed; moreover, 25 WRKY TFs were differentially expressed in *G. thurberi* leaves, and most of them were up-regulated. The results obtained were in agreement with the previous studies, which elucidated the WRKY TFs in *Arabidopsis thaliana*, which played an important role in enhancing cold stress (Wang *et al.* 2011). Interestingly, gene3257 encoding a WRKY TFs displayed opposite expression trends at 6 and 12 h time point in our data. The expression of this gene was inhibited at early stages, while it was induced after 12 h of cold treatment, implying that this gene might be functioning in different ways at different time points.

The NAC TFs are involved in many aspects of plant growth, development and response to abiotic stress (Chen *et al.* 2016). The NAC family TFs, which were closely associated with ABA signal pathways, showed significant up-regulation in the leaf tissues of *G. thurberi* under cold stress. Moreover, *OsNAC5* a NAC transcription factor was significant in enhancing cold, drought and salinity stress tolerances in rice plants, by regulating downstream targets associated with accumulation of compatible solutes, H_2_O_2_ and MDA (Song *et al.* 2011). Furthermore, it has been reported that *bHLH* TF do act as positive regulators of the CBF pathway and conferred cold tolerance in plants (Niu *et al.* 2017). Moreover, overexpression of *NtbHLH123* in tobacco reduced the electrolyte leakage, MDA contents, H_2_O_2_ and ROS accumulation under cold stress, thus enhancing cold tolerance (Zhao *et al.* 2018). We found that the expression of 32 *bHLH* genes were both altered after 6 and 12 h of cold treatment in *G. thurberi*. Among these genes, were 14 and 18 up- and 18 down-regulated *bHLH* genes, respectively, in the leaf tissues of *G. thurberi* under cold stress condition. The results suggested that the cold-induced *bHLH* genes might be playing a critical role in the regulation of the rhythm of *G. thurberi* under cold condition.

Due to the amount of *MYB* TFs found in *A. thaliana* (Stracke *et al.* 2001), rice (Katiyar *et al.* 2012), tomato (Meng *et al.* 2015), maize (Rabinowicz *et al.* 1999) and cotton (Salih *et al.* 2016), a lot of researches have been focused on its role in transcriptional regulation and impact on the physiological function. For example, overexpression of rice *MYBS3* significantly enhanced cold tolerance in transgenic rice (Su *et al.* 2010). In this research, 17 MYB genes were found to exhibit differential expression pattern under cold stress treatment. These results indicated that *ERF*, *TCP*, *WRKY*, *NAC*, *bHLH* and MYB TFs were involved in plant responses to various stresses, and further suggested that cold stress might have a common molecular mechanisms with other abiotic stress.

As a ubiquitous second messenger in cellular signal transduction, calcium (Ca^2+^) acts as a mediator of stimulus response, coupling in the regulation of diverse cellular functions (Feng *et al.* 2013). For instance, a quantitative trait locus (QTL) *COLD1* encoding a regulator of G-protein signalling has been found to confer chilling tolerance in *japonica* rice under cold stress (Ma *et al.* 2015), because it facilitated the activity of the Ca^2+^ channel which is responsible for sensing low temperature by increased Ca^2+^concentration (Györke and Györke 1998). Moreover, numbers of the DEGs involved in signal transduction were related to the calcium-dependent signal pathway. For instance, calcium-dependent protein kinases (CDPKs), a kind of serine/threonine protein kinase, which is activated by calcium signal, and are involved in carbon and nitrogen metabolism, ion and water transmembrane transport, growth and development regulation as well as response to anti-abiotic stress (Kumar *et al.* 2004). The expression of *gene10339* was significantly up-regulated under cold stress in *G. thurberi*. In addition, the expression of nine CIPKs and 15 CMLs genes was altered by cold stress, and most of them were up-regulated, revealing that the proteins encoded by the genes could be playing an important role in detection and transmission of the cold signal. The results were consistent with previous studies which reported that overexpression *ShCML44* gene enhanced cold, drought and salinity stresses tolerance in *Solanum lycopersicum* transgenic plants (Munir *et al.* 2016). The expression of three *CML*44-like genes was highly increased in leaves at two time points of cold treatment, which suggested that these genes play a significant role in the Ca^2+^ signal transduction pathway in cotton and might be contributing to the increased tolerance to low temperatures.

Plants could perceive and respond to stress rapidly via signal transduction pathways mediated by phytohormones. Most of the DEGs related to plant hormone signal transduction were up-regulated in all the stages under cold treatment, suggesting that plant hormone signal transduction plays a core role in response to cold stress. In particular, ABA plays a role in signal detection in response to drought, cold, osmotic stress and pathogen infection, in turn triggering various changes in plant physiology and development processes, resulting in adaptation to biotic and abiotic stresses (Mittler and Blumwald 2015). Several ABA biosynthesis-related genes, including five 9-*cis*-epoxycarotenoid dioxygenase (NCED) and three abscisic aldehyde oxidase (AAO) genes, were induced under cold stress. Moreover, ABA does induce H_2_O_2_ by activating the nicotinamide adenine dinucleotide phosphate (NADPH) oxidases such as *AtrBOHD* and *AtrBOHF* in *Arabidopsis* (Alqurashi *et al.* 2017).

Plants have evolved a complex molecular mechanism network to adapt to various abiotic stresses such as cold, drought and salt, by enhancing their adaptability through the mobilization of various stress genes, such as the *LEA* genes (Magwanga *et al.* 2018), *MATE* genes (Lu *et al.* 2019) among others. Moreover, plant stress responses must be coordinated with growth and development, it is important to understand the crosstalk between stress signalling pathways and hormonal as well as growth and development signalling pathways (Kim *et al.* 2015). The cold response pathway ICE-CBF-COR is a best-known defence mechanism to cold stress, which includes the core component inducer of CBF expression (ICE), C-repeat binding factors (CBF) transcriptional factor and other diverse proteins such as the cold-regulated proteins. As master regulators of CBF expression, *ICE* binds to the promoter of the *CBF* genes, and positively induces the expression of *CBF* genes (Chinnusamy *et al.* 2003). Furthermore, overexpression of the *ICE1* gene enhanced plant tolerance to cold stress, whereas wild plants were hypersensitive to cold stress (Xiang *et al.* 2013, 2017). The *ICE2* gene is highly homologous to *ice1*, it has been found to have the ability to trigger the expression of *CBF1* (Fursova *et al.* 2009). Overexpression of *ICE2* resulted in an increased survival rate in transgenic plants after freezing treatment (Kurbidaeva *et al.* 2014). Moreover, two *ICE1* genes (gene8003 and gene3257) and two *ICE2* genes (gene16424 and gene24288) were identified in *G. thurberi*. Although the expression levels of the two *ICE2* genes were not obviously different under cold treatment and control samples, but the VIGS results showed that the *ICE2* genes are important for cold tolerance in cotton. Moreover, as the three important transcriptional factors involved in cold response pathway in *Arabidopsis*, CBF1 and CBF3 are negatively regulated by CBF2 (Zhou *et al.* 2011). Although previous studies have shown that the *CBF4* gene, a novel homolog of *CBF*/*DREB1* genes from *Arabidopsis*, was initially induced under drought stress (Karimi *et al.* 2015). Interestingly, the expressions of the *CBF1* and *CBF3* genes were neither up- nor down-regulated under cold treatment; however, the *CBF2* and *CBF4* genes were activated by cold stress, especially the expression of *CBF4* gene was highly up-regulated when *G. thurberi* seedlings were exposed to low temperature. In addition, *RAP2.6*, an *Arabidopsis* AP2/ERF family member, has been reported to function in plant response to ABA, and different stress conditions such as salinity, cold and osmotic stresses (Imran *et al.* 2018). In this study, we found that the expression level of *RAP2.7* (gene6353) from *G. thurberi* was up-regulated in two stages after exposure to cold stress, suggesting that *RAP2.7* gene may be a key gene involved in the CBF-regulated network. As CBF target genes, the COR genes have a significant role in enhancing plant tolerance to cold stress (Dong *et al.* 2014). Furthermore, several *COR* genes were induced under the cold condition in this study. All these results indicated that there might be a different cold regulating pathway in cotton and *CBF4* and *RAP2.7* genes could be the key cold candidate genes in cotton. A well-studied cold response pathway is the ABA-dependent signalling pathway, which mainly contains the pyrabactin resistance/pyrabactin resistance-like/regulatory component of the abscisic acid receptor (PYR/PYL/RCAR) family as ABA receptors, negative/positive regulators such as type 2C protein phosphatase (PP2C) and SNF1-related protein kinase 2 (SnRK2) and a series of other TFs (Zhu 2016). In rice, the concentration of endogenous ABA increased under cold stress, ABA bind to the PYR/PYL/RCAR, which then interacts and inhibits PP2C, resulting in the activation of SnRK2. The activated SnRK2 phosphorylate effector proteins including TFs (AREB, ABF) and downstream stress responsive genes were activated to improve plant tolerance against low temperature environment (Sah *et al.* 2016). In rice, overexpression of *OsPYL3* and *OsPYL9* enhanced rice tolerance to chilling stress (Tian *et al.* 2015). Moreover, all of the three *PYL4* and one *PLY8* genes were up-regulated under cold stress. The up-regulation of *PYL4* and *PYL8* genes in addition to the *PP2C* gene (ABA biosynthesis genes), indicated that the ABA signalling pathway is integral in cold stress tolerance in plants.

In confirming the role of *CBF4* and *ICE2* genes through VIGS, the VIGS plants ability to tolerate cold stress was significantly reduced, as evident by higher levels of MDA and significant reduction of PRO and SOD, the two antioxidant enzymes evaluated. When plants are exposed to any form of stress, the delicate balance of reactive oxygen production and elimination becomes altered, leading to excessive accumulation of ROS within the plant cells (Dong *et al.* 2019).

## Conclusion

In conclusion, we presented the first comprehensive transcriptome data from *G. thurberi*, a wild diploid cotton species that have adapted to local temperature climate and exhibits strong tolerance to low temperature. The transcriptome revealed that a total of 4226 DEGs were discovered to be involved in cold stress tolerance in leaves. Furthermore, we identified several DEGs related to hormones, Ca^2+^ and ROS, which were involved in different cold response pathways suggesting the complex responses of *G. thurberi* towards cold stress. Moreover, RT-qPCR analysis and VIGS showed that the *CBF4* and *ICE2* genes could be the novel genes in *G. thurberi*, which are responsible for its cold stress tolerance. The virus-induced gene silenced plants (VIGS plants) ability to tolerate cold stress effect was significantly reduced compared to their wild types, as evident by higher levels of MDA and a significant reduction in PRO and SOD concentrations. Our findings provide an overall picture of the regulatory network in response to cold stress in *G. thurberi*. These COR-related genes could be targeted as potential candidates for further study and be vital in developing more cold stress tolerance cotton genotypes.

## Sources of Funding

This research was funded by the Natural Science Foundation of China (31601352).

## Contributions by the Authors

C.X., R.O.M., W.K. and F.L. designed the experiment, implemented and collected the data. C.X., Y.X., and R.O.M. analysed the results and prepared the manuscript. C.X., R.O.M., Z.Z., X.X.W., Y.H., Y.W., Y.Z., F.L., W.K. revised the manuscript. All authors reviewed and approved the final manuscript.

## Supporting Information

The following additional information is available in the online version of this article—

**Figure S1.** Distribution’s network of reactive oxygen species (ROS) 692 genes in chromosomes of *Gossypium hirsutum*, *G. raimondii* and *G. arboretum.*

**Table S1.** Genes of mapped reads assemble and annotation of *Gossypium thurberi.*

**Table S2.** Differentially expressed genes (DEGs) of *Gossypium thurberi.*

**Table S3.** Primers list of transcripts for real-time RT-PCR.

## Supplementary Material

plz045_suppl_Supplementary_Figure_S1

plz045_suppl_Supplementary_Table_S1

plz045_suppl_Supplementary_Table_S2

plz045_suppl_Supplementary_Table_S3
