# Supplementary figures and images for: Comparative transcriptome, physiological and biochemical analyses reveal response mechanism mediated by CBF4 and ICE2 in enhancing cold stress tolerance in Gossypium thurberi
Source: AoB Plants. 2019 Aug 9;11(6):plz045. doi: 10.1093/aobpla/plz045 (PMC6863471; doi:10.1093/aobpla/plz045)

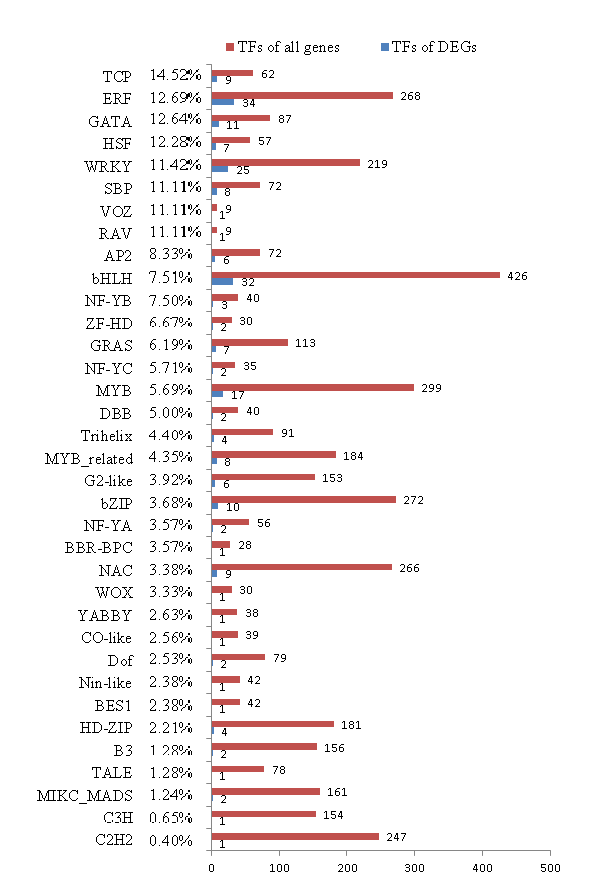

Supplement: plz045_suppl_Supplementary_Figure_S1 [file plz045_suppl_supplementary_figure_s1.png]
