# Supplementary material for: Comparative transcriptome, physiological and biochemical analyses reveal response mechanism mediated by CBF4 and ICE2 in enhancing cold stress tolerance in Gossypium thurberi
Source: AoB Plants. 2019 Aug 9;11(6):plz045. doi: 10.1093/aobpla/plz045 (PMC6863471; doi:10.1093/aobpla/plz045)
Supplement: plz045_suppl_Supplementary_Table_S3 [file plz045_suppl_supplementary_table_s3.docx]

Table S3:. Primers list of transcripts for real-time RT-PCR

| **gene ID** | **F** | **R** |  |
| --- | --- | --- | --- |
| gene26687 | AGGGTTGTTCGATTTCAT | TTCCTCTGCCTTTGTCTC |  |
| Gene8003 | CCCATCTACACAAACGAA | ACATACAGGAAGCAGTCC |  |
| Gene6664 | AGGGTTAATTGATTTGGC | ATTTCATCTTGGTCTGGC |  |
| Gene29039 | CGATGCTGTTGCTGTGTC | TCCTCTGGTTTTATGCCC |  |
| Gene35298 | CGTAGAGGCAAAGAAAGTA | ATAAGGATTAGCCACCAGA |  |
| Gene3257 | CCTCTCTTTCAAACCCCG | GCTTCTTCAACTCCCGCT |  |
| Gene16424 | CTCAAATCTGCCTCTGCT | CGAACTTCTTCTTCCCAA |  |
| Gene43084 | AAAGCATTGCGATGGTCC | TACTCCCGCCGTTTACCC |  |
| Gene8413 | CATCTCGGCTACCACCAC | TCATCACCTTCCCCTTCT |  |
| Gene39126 | ATGAGCAGCCCAACAAAG | TGCCAGCGAGTGAAGACG |  |
| Gene24288 | GCCTTTTCTATTTCTGC | ATCCCCTCACTCTCCTC |  |
| Gene3704 | TCAACGAGTCCAAGCCCC | CGCACATAGAATCCGCCA |  |
| Gene12713 | TCCTTTGCCTTCTCAGC | GCCACAACAAGACCATC |  |
| Gene8330 | TGTTTCTTTGGTTCCCG | ACAGTCCTTAGGCGTTT |  |
| Gene14397 | TTGGTTGCGGCTGTGGTA | AGCGGGCTTAGATGAGGG |  |
| Gene6353 | TGGCAAATAAAGGGAGG | ACTATGAGTGCGGGGAT |  |
| Gene27708 | GGAGTTGCCTTGAGTGTTGT | TTGTTGCCTGGTGAGTTCTT |  |
| Gene1426 | ATTGTTTCTGAGGAGGGG | ATCTGGTATCAAGGGTGG |  |
| Gene34136 | GAAAACTACTCAATCCCAAC | GAAACTAAAGCATTCTCCAC |  |
| gene9553 | GAAGCTGTTTTTGTGCCTCG | TCTGCTGACATCGTTTTTGC |  |
| Gene26027 | ATTTGACCCCAGAACACTAC | TCCCGATGAAAAACATTAGC |  |
| Gene27706 | GTGCGGTTCATTTTACTCCA | GGTCCACCACATTCTTCCCT |  |
